# Parental perceptions of child and family mental health and behavioural adjustment following a mass school shooting in Serbia

**DOI:** 10.3389/fpubh.2026.1847185

**Published:** 2026-10-07

**Authors:** Miodrag Stankovic, Aleksandra Stojanovic

**Affiliations:** 1 Medical Faculty, University of Nis, Niš, Serbia; 2 Centre of Mental Health Protection, University Clinical Centre of Nis, Niš, Serbia

**Keywords:** caregiver perceptions, child mental health, community mental health, parental distress, psychological stress, risk perception, school violence

## Abstract

**Objective:**

Study examined parental perceptions of their own and children’s mental health and behavioural responses among parents who were not directly or indirectly exposed to the 2023 school shooting in Serbia.

**Methods:**

The study sample included 1,927 parents. The questionnaire assessed pre-event child mental health characteristics and support utilisation, parental threat perception, emotional distress, functional disruption of daily routines, observed child emotional and behavioural changes, help-seeking behaviour, and perceived access to mental health support.

**Results:**

Prior to the shooting, 11.7% of children had a psychiatric diagnosis. Following the shooting, 85.2% of parents reported moderate-to-high concern for child safety, 38.7% reported marked difficulty sending children back to school, and 48.0% reported difficulty controlling intrusive thoughts about child victimization. Most parents observed no emotional or behavioural changes in their children; this finding should be interpreted as an absence of parent-observed changes rather than clinically defined resilience, given the reliance on parental report alone. However, children with pre-existing difficulties showed significantly higher rates of post-event worsening. Mothers reported greater distress and impairment than fathers across all domains. Post-event utilisation of formal mental health services was low (3.8% for children, 2.9% for self).

**Conclusion:**

Findings suggest that mass school shootings may be associated with substantial parental distress and functional disruption even without direct or indirect exposure. The gap between epidemiological estimates and low pre-event diagnosis rates raises concern about possible under-recognition of child mental health needs. Low help-seeking highlights the importance of accessible, non-stigmatising psychosocial support, particularly school-based psychoeducation, rather than formal clinical treatment alone. Findings should be interpreted in light of methodological limitations.

## 1 Introduction

A school shooting is defined as an incident in which a current or former student of an educational institution intentionally uses a firearm to injure or kill at least one student or staff member on school premises during school-related activities (1). School shootings occur in environments traditionally and symbolically associated with childhood safety and institutional protection, and their psychological impact chalenge perception and attitudes about safety (2).

Between 1997 and 2022, there were 1,453 school shootings in the United States, with incidence accelerating markedly in the past five years (3). According to the K-12 School Shooting Database, which tracks all firearm incidents on school grounds since 1966 regardless of casualties, 2023 recorded the highest annual number ever (*n* = 349), followed by 2024 with 330 incidents, the second-highest on record (4). Definition limited to incidents resulting in at least one injury or death during school hours, 40 shootings were recorded in 2024 and 18 in 2025, maintaining historically high levels of such incidents (32).

On May 3, 2023, Serbia experienced its first fatal school shooting at “Vladislav Ribnikar” Elementary School in Belgrade, in which a 13-year-old student killed nine pupils and one school security officer. The following day, a separate mass shooting occurred in the villages of Dubona and Malo Orasje, perpetrated by an adult assailant; whilst that incident involved school-related locations during non-school hours, it did not meet definitional criteria for a school shooting and is therefore analytically distinct. The temporal proximity and extensive media coverage of both events contributed to a nationwide atmosphere of threat and insecurity. It is consistent with evidence that mass shootings and collective traumas can affect perceived safety and mental health well beyond directly exposed individuals (5–9).

Existing research on school shootings has predominantly focused on directly exposed survivors and bereaved families, documenting profound and enduring consequences including PTSD, depression, prolonged grief, and functional impairment across age groups (2, 5, 10–12). In contrast, considerably less attention has been paid to parents whose children were not directly or indirectly exposed, despite evidence that mass violence can exert substantial psychological effects at the population level (5–7). Such indirect effects are often mediated by perceived threat, media exposure, and disruption of core assumptions about safety, rather than by objective exposure alone (13, 14).

Parents represent a particularly important population in this context. Beyond processing their own distress, parents play a central role in regulating children’s reactions to threat, maintaining daily routines, and parent–child communication (14). Prior research indicates that perceived inability to protect one’s child in the face of unpredictable and lethal danger may generate intense peritraumatic distress and sustained hypervigilance (15–17). Even following mass violence events in which children are not directly harmed, parents have reported clinically significant levels of PTSD symptoms, depression, and functional impairment persisting well beyond the acute phase (18). Disruptions in parental functioning may adversely affect children’s adjustment through bidirectional parent–child communication (8, 15), a dynamic that may occur even when children are not directly exposed to violence.

The academic literature on the societal impact of mass shootings on parental emotional, cognitive, and behavioural responses in indirectly or non-exposed populations remains limited, particularly outside the United States (6, 19). Little is known about how such events influence parental perceptions of child safety, everyday functioning, help-seeking behaviour, and perceptions of access to mental health support in contexts where school shootings are historically unprecedented (10).

The present study draws on four complementary theoretical frameworks to understand how a school shooting may affect parents without direct exposure. First, the cognitive model of threat appraisal (20) proposes that subjective perception of threat, rather than objective danger, is the primary driver of psychological stress responses. Parents who appraise schools as dangerous following a shooting may develop intrusive cognitions, hypervigilance, and avoidant behaviours even without personal involvement. Second, the peritraumatic distress model (17) describes how acute helplessness upon learning of a traumatic event predicts later functional impairment, particularly when individuals cannot protect those they care for. A mechanism is directly relevant to the parental experience of school shootings. Third, the stress–vulnerability model (15) emphasises that children with pre-existing mental health difficulties are at disproportionate risk of deterioration following community-level stressors, and that parental distress may further compromise their recovery through bidirectional parent–child processes (8). Fourth, Family Stress Theory (21, 22) proposes that crisis events place demands on the family system that exceed its routine coping resources, generating an accumulation of concurrent stressors: anxiety about safety, disrupted routines, and the burden of explaining collective trauma to children, that may disrupt multiple functional domains simultaneously. Together, these frameworks predict that non-exposed parents may experience genuine psychological and functional consequences across multiple domains, that mothers may report higher distress than fathers (20), and that children with prior vulnerabilities constitute an identifiable at-risk subgroup. In that context, study addresses the following research questions: (RQ1) What proportion of parents report pre-event mental health diagnoses and service use for their children, and how do these rates compare to epidemiological estimates? (RQ2) To what extent do non-exposed parents report elevated threat perception, emotional distress, and functional disruption following the shooting? (RQ3) Are there significant differences between mothers and fathers in safety concerns, functional impairment, and cognitive–emotional responses? (RQ4) What parent- and child-level factors independently predict difficulty sending children back to school? (RQ5) What are the rates of post-event help-seeking and perceived access to mental health support in this population?

By addressing these domains, the study aims to advance understanding of the indirect, population-level psychological impact of school shootings on parents and families and to inform preventive and post-crisis responses within educational and public health systems.

## 2 Methods

The present study uses the same dataset and has also been used in a related study examining different research questions and outcome variables (23).

### 2.1 Study design

The study employed a repeated cross-sectional design comprising two independent samples collected at different post-event time points. The first phase served as a feasibility pilot, its primary purpose was to assess instrument adequacy and determine whether the research question warranted a full-scale national investigation. and does not constitute a longitudinal study. The two samples are independent, the two phases are not compared, and Phase 1 data are not reported separately, and no cross-phase comparisons are reported in this manuscript.

Phase 2 was conducted 12 months after the shooting with a nationwide sample distributed through the Ministry of Education. Both phases used the same core instrument and identical response formats; however, several items were modified in the final version following an institutional requirement.

### 2.2 Sampling and recruitment

#### 2.2.1 Phase 1: pilot study

Phase 1 was conducted approximately 60 days post-event as a regional pilot survey. Participants in the feasibility pilot phase were recruited primarily through convenience sampling. The survey link was disseminated via direct email, social media, and personal communication with school psychologists and school administrators, who were asked to forward it to parents within their school communities. An element of snowball recruitment was present in that some participants may have shared the link further, This was not formally tracked. Participation was voluntary, and no incentives were offered.

#### 2.2.2 Phase 2: nationwide study

In the second phase, the survey was distributed nationwide via the Ministry of Education of the Republic of Serbia’s official internal communication system. The Ministry forwarded the survey invitation to all primary and secondary schools, instructing school administrations to inform parents about the study and invite them to participate voluntarily. Parents accessed the survey through an anonymous online link.

Because the survey was distributed through institutional channels without direct contact with individual parents, the total number of recipients could not be determined and a formal response rate could not be calculated. This is an inherent limitation of the institutional distribution method.

### 2.3 Participants

Eligible participants were parents or primary caregivers of children in Serbian elementary or secondary schools. Exclusion criteria were direct or indirect exposure to the shooting events, inability to provide informed consent, and insufficient Serbian proficiency. Respondents reporting either type of exposure were excluded from subsequent analyses. Of the 1,969 respondents, 42 were excluded due to direct or indirect exposure to the events, resulting in a final sample of 1,927 participants.

To contextualise the sample, key sociodemographic characteristics are compared with national demographic indicators in Table 1. The sample is substantially dominated by mothers (86%), which limits the generalisability of findings to fathers and should be considered when interpreting gender comparisons.

**Table 1:** Sample characteristics and demographics (*N* = 1,927).

Characteristic	Category	*n*	%
Respondent role			
	Mother	1,665	86.4
	Father	238	12.4
	Other caregiver (relative/step-parent/foster)	24	1.2
Parent sex			
	Female	1,679	87.1
	Male	248	12.9
Parent age (years)	Mean (SD); range		42.6 (6.2); 18–70
Residence			
	Urban	1,109	57.6
	Rural	536	27.8
	Suburban	282	14.6
Education level			
	Primary school or less	84	4.4
	Secondary school	833	43.2
	College/higher vocational education	258	13.4
	University degree	543	28.2
	Master’s degree	150	7.8
	Doctoral degree	30	1.6
	Other/unspecified	29	1.5
Employment status			
	Full-time employment	1,430	74.2
	Part-time employment	74	3.8
	Unemployed/reduced working hours	142	7.4
	Homemaker	262	13.6
	Retired	16	0.8
	Student	3	0.2
Household structure			
	Two-parent household	1,599	83.0
	Single-parent household	286	14.8
	Other configurations	42	2.2
Index child age (years)	Mean (SD); range		13.1 (3.2); 5–20
Index child sex			
	Female	968	50.2
	Male	959	49.8
School type (index child)			
	Public school	1,913	99.3
	Private school	2	0.1
	Specialised school	3	0.2
	Other	9	0.5
Prior diagnosis (index child)			
	Any reported diagnosis	226	11.7
	No diagnosis reported	1,701	88.3

*N* = 1,927 represents the final sample after exclusion of 42 respondents (2.1%) reporting direct or indirect exposure to the shooting events. Index child = the child identified by the parent as showing the most pronounced response, or the eldest school-age child if no differential response was observed.

### 2.4 Survey

The survey, which had 50 items, assessed parental emotional and cognitive responses, perceptions of safety, access to psychological support, and changes in daily life after the mass school shooting.

Items assessing parental responses to school shootings were developed specifically for this study. The questionnaire structure and response formats were adapted from a Caregiver Needs Survey from the Global Autism Public Health Initiative for Autism Speaks, previously used by the first author during the COVID-19 pandemic (19). Adaptations included: (a) replacing all autism- and pandemic-specific content with items relevant to mass violence and school safety; (b) revising response anchors to reflect parental threat perception and functional disruption; and (c) adding domains specific to the post-shooting context (e.g., help-seeking behaviour, school return difficulty, and perceived institutional support). The adapted instrument was not psychometrically validated prior to use in this study. The use of single-item ordinal indicators for several constructs is acknowledged as a methodological limitation (see Strengths and Limitations). Whilst the general format and scaling were retained, all items were newly written and adapted to the context of mass violence and school shootings.

The questionnaire was conceptually organised into nine broad thematic areas. These included: (1) sociodemographic characteristics; (2) exposure to the event; (3) direct and indirect impact of the event; (4) information and media exposure; (5) continuity and accessibility of support and care services; (6) parental and family functioning following the event; (7) barriers to and facilitators of psychological recovery and adaptation; (8) development of psychological symptoms and difficulties in children and parents; and (9) attitudes toward risk mitigation and prevention of future adverse outcomes.

### 2.5 Procedure

Data collection in Phase 1 took place in July 2023, about 60 days after the school shooting. This phase was conducted as a local pilot study. Phase 2 data collection was carried out 12 months after the event, from May through the end of November 2024. Phase 2 included participants from all regions of Serbia.

Participants provided electronic informed consent before proceeding; only those who consented could access the survey items. Information regarding voluntary participation, anonymity, and data confidentiality is reported in Section 2.6.

The pilot questionnaire consisted of 50 items in Serbian and was designed to be completed in approximately 20 min.

Pilot form questionnaire link: https://docs.google.com/forms/d/e/1FAIpQLSejStlDOPEv9egDM21zNjXpKXBc5G_l_uQPua8e_9o_uJzDXQ/viewform?usp=sf_link.

The final form: https://docs.google.com/forms/d/e/1FAIpQLScbWK13o5aeCjW4tIZDFItsgAvF7rZTmtKZuk8KnHjO42fVUQ/viewform?usp=sf_link.

Following institutional review by the Ministry of Education, several items from the pilot version were removed due to concerns related to public sensitivity. Specifically, questions addressing hypothetical scenarios involving the use of lethal force by school police officers were excluded. One such item asked whether a school police officer would be permitted to use lethal force against an armed child who refused to surrender and posed an immediate threat. The revised instrument, approved by the Ministry of Education, also contained 50 items in Serbian. In addition, based on recommendations from the Ministry, the questionnaire was administered exclusively to parents and caregivers.

### 2.6 Data analysis

The questionnaire was initially structured around nine predefined thematic areas. The analytical strategy was to organise results into broader conceptual groupings. These groupings were derived during data analysis by integrating conceptually related variables across domains in order to enhance interpretability and analytical coherence.

Participants who reported direct or indirect exposure to the school shooting events were excluded from inferential analyses.

Data were analysed using descriptive and inferential statistical methods. Group differences between mothers and fathers were examined using independent-samples t-tests for approximately normally distributed continuous variables, and Mann–Whitney *U* tests for ordinal variables. Categorical variables were compared using chi-square tests. Spearman rank-order correlations were used to examine associations between ordinal variables. For exploratory analyses of residence-type differences, one-way ANOVA and ANCOVA were applied treating ordinal scores as approximately interval-level. This is acknowledged as an assumption and primary conclusions rest on nonparametric methods. For the logistic regression predicting marked difficulty sending the child back to school, the outcome was dichotomised into scores 4–5 versus 1–3. This approach is interpretable but sacrifices information and findings should be regarded as hypothesis-generating. Effect sizes (Cohen’s *d*, Cramer’s *V*, odds ratios, and Spearman’s *r*) and 95% confidence intervals are reported throughout. Given the large sample size (*N* = 1,927), statistical significance is expected even for small effects. Findings are therefore interpreted with emphasis on effect magnitude rather than *p*-values alone. Effect sizes (Cohen’s *d* and Cramer’s *V*) and 95% confidence intervals were reported where applicable. Composite scores were calculated as the mean of conceptually related items.

### 2.7 Ethics statement

The study was approved by the Ethics Committee of the University Clinical Center Niš (No. 19486/11, July 7, 2023) and conducted in accordance with the Declaration of Helsinki. Participation was entirely voluntary; participants were informed that they could withdraw at any time without consequence. All data were collected anonymously and no personally identifying information was stored. Participants received written information about the study aims and procedures before providing active electronic informed consent. Data were stored securely and accessed only by the research team.

## 3 Results

The questionnaire originally contained nine conceptual areas. For reporting, results are organised into seven theoretically informed thematic domains, addressing research questions RQ1–RQ5. Each domain integrates conceptually related items from the original instrument. Findings are presented in sequence below, with inferential analyses reported within each domain.

### 3.1 Sample characteristic and demographics

From the full dataset (*N* = 1969), 42 respondents (2.13%) reported direct or indirect exposure to the events. These responses were excluded from inferential analyses. The final sample comprised *N* = 1,927 parents or caregivers without direct or indirect exposure to the shooting events (see Section 2.3). Full sociodemographic characteristics are presented in Table 1.

In terms of caregiving role, most participants were mothers (86.4%), followed by fathers (12.4%), with the remainder comprising other caregivers such as grandparents, step-parents, and foster caregivers (1.2%).

The mean parental age was 42.6 years (SD = 6.2, range 18–70).

Most respondents lived in urban areas (57.6%), with smaller proportions in rural (27.8%) and suburban areas (14.6%). The most common educational levels were secondary school (43.2%) and university degree (28.2%). Most respondents were employed full-time (74.2%), with homemakers comprising 13.6%. Full frequencies are provided in Table 1.

Most households were two-parent (83.0%), with 14.8% single-parent and 2.2% other configurations.

The mean age of the index child was 13.1 years (SD = 3.2, range 5–20). The sex distribution was balanced (female 50.2%, male 49.8%). Nearly all children attended public schools (99.3%). Most children had no previously reported diagnosis (88.3%), whilst 11.7% had at least one prior psychiatric or psychological diagnosis. Full details are provided in Table 1.

### 3.2 Parental perceptions and responses following the shooting

#### 3.2.1 Domain 1. Pre-event child mental health and support context

Prior to the event, 11.7% of index children had at least one reported psychological or psychiatric diagnosis, whilst the vast majority of parents reported no use of mental health services for their child (93.9%) or themselves (94.1%) in the preceding 6 months. Among parents reporting pre-existing child difficulties, schools were informed in approximately two-thirds of cases. The most frequently reported psychiatric condition among children was anxiety disorder (1.2%). Full details are provided in Table 2.

**Table 2:** Pre-event child mental health and support context (*N* = 1,927).

Indicator	*n*	%
Pre-event child mental health		
Child had any prior psychiatric/psychological diagnosis	226	11.7
Most frequently reported: anxiety disorder	23	1.2
No child mental health service use (preceding 6 months)	1,810	93.9
No parent mental health service use (preceding 6 months)	1,813	94.1
School awareness and support (full sample)		
School informed about child difficulties (among those with difficulties, *n* = 143)	90	62.9
School NOT informed (among those with difficulties)	53	37.1
School provided additional psychological/pedagogical support: Yes	182	9.4
School provided additional support: No (but needed)	141	7.3
School support: No need reported	1,604	83.2
Barriers to obtaining school-based support (any)	93	4.8
Perceived contributors to child difficulties (pre-event)		
Any perceived contributor endorsed	134	7.0
School bullying	68	3.5
Cyberbullying	22	1.1
Witnessing peer bullying	22	1.1

Percentages for school awareness and support items (rows 3–8) are calculated from the full sample (*N* = 1,927). Percentages for school-informed/not-informed are calculated from those reporting child difficulties (*n* = 143). All figures in table represent the Phase 2 analytic sample.

In inferential analyses, prior child diagnosis was significantly associated with post-event worsening of child mental health problems (*χ*^2^ = 27.02, *p* < 0.001, Cramer’s *V* = 0.12), indicating a small-to-moderate statistical association between pre-existing vulnerability and post-event deterioration.

Among respondents indicating that their child had difficulties (i.e., excluding the response “the child has no difficulties”), 62.9% reported that the school was informed, whereas 37.1% reported that the school was not informed (*n* = 53). Before the incident, 9.4% of parents reported that the school provided additional psychological/pedagogical support, whilst 7.3% reported that support was not provided. Some barriers to obtaining school-based support were reported by 4.8% of parents. Perceived contributors to the onset/worsening of the child’s condition were endorsed by 7.0%, most commonly school bullying (3.5%), cyberbullying (1.1%), and witnessing peer bullying (1.1%). Pre-event child mental health and support context data are provided in Table 2.

#### 3.2.2 Domain 2. Parental threat perception and safety concerns

Addressing RQ2 and RQ3: following the mass shootings, parents reported substantially greater concern for child safety than for their own safety. Moderate-to-high concern about personal safety was reported by 61.5% of parents. Concern about the child’s safety was substantially higher: 85.2%, including 68.9% reporting great concern. Moderate-to-high concern about losing a loved one was reported by 81.7%, and moderate-to-high concern about becoming a victim by 62.3%. Using ordinal comparisons, mothers reported significantly greater concern for child safety than fathers (median = 5 vs. 4), as demonstrated by a Mann–Whitney *U* test (*U* = 259,472.5, *p* < 0.001, *r* = 0.15). Similar, though slightly smaller, differences were observed for concern about personal safety (*U* = 252,948.5, *p* < 0.001, *r* = 0.14). Detailed frequencies are in Table 3.

**Table 3:** Parental threat perception, functional difficulties, and cognitive–emotional responses following the shootings (*N* = 1,927).

Item	Category	*n*	%
Domain 2. Parental threat perception and safety concerns			
Concern for personal safety	Low (1–2)	741	38.5
	Neutral (3)	510	26.5
	High (4–5)	676	35.1
Concern for child’s safety	Low (1–2)	286	14.8
	Neutral (3)	313	16.2
	High (4–5)	1,328	68.9
Concern about losing a loved one	Low (1–2)	353	18.3
	Neutral (3)	570	29.6
	High (4–5)	1,004	52.1
Concern about becoming a victim	Low (1–2)	726	37.7
	Neutral (3)	485	25.2
	High (4–5)	716	37.2
Domain 3. Parental functional difficulties			
Difficulty sending child back to school	Easy/very easy (1–2)	500	25.9
	Neutral (3)	682	35.4
	Difficult/very difficult (4–5)	745	38.7
Difficulty going to work	Easy/very easy (1–2)	855	44.4
	Neutral (3)	646	33.5
	Difficult/very difficult (4–5)	426	22.1
Difficulty allowing child peer time outside home	Easy/very easy (1–2)	791	41.0
	Neutral (3)	716	37.2
	Difficult/very difficult (4–5)	420	21.8
Difficulty engaging in relaxing/enjoyable activities	Easy/very easy (1–2)	631	32.7
	Neutral (3)	852	44.2
	Difficult/very difficult (4–5)	444	23.0
Difficulty maintaining daily routines	Easy/very easy (1–2)	691	35.9
	Neutral (3)	838	43.5
	Difficult/very difficult (4–5)	398	20.7
Difficulty maintaining healthy lifestyle habits	Easy/very easy (1–2)	865	44.9
	Neutral (3)	756	39.2
	Difficult/very difficult (4–5)	306	15.9
Difficulty resisting constant checking of updates	Easy/very easy (1–2)	476	24.7
	Neutral (3)	788	40.9
	Difficult/very difficult (4–5)	663	34.4
Domain 4. Parental cognitive–emotional processing			
Difficulty not thinking child could be a victim	Easy/very easy (1–2)	371	19.3
	Neutral (3)	632	32.8
	Difficult/very difficult (4–5)	924	48.0
Difficulty not thinking child could perpetrate an attack	Easy/very easy (1–2)	1,409	73.1
	Neutral (3)	231	12.0
	Difficult/very difficult (4–5)	287	14.9
Difficulty helping child express feelings	Easy/very easy (1–2)	930	48.3
	Neutral (3)	491	25.5
	Difficult/very difficult (4–5)	506	26.3
Difficulty explaining killing is not normal	Easy/very easy (1–2)	1,560	81.0
	Neutral (3)	192	10.0
	Difficult/very difficult (4–5)	175	9.1

Scores 1–2 = low concern/easy; score 3 = neutral/moderate; scores 4–5 = high concern/difficult. n for score 3 (Neutral) is derived as N minus scores 1–2 and 4–5. Column totals may not sum to 100% due to rounding.

When residence type was examined, one-way ANOVA (treating ordinal concern scores as approximately interval, as justified in Section 2.5) showed no significant differences across urban, suburban, and rural settings (*F*(2,1924) = 2.64, *p* = 0.071, *η*^2^ = 0.003, negligible effect).

In ANCOVA models adjusting for parent gender, age, household structure, and residence, being a mother (*β* = 0.53, *p* < 0.001) and urban residence (*β* = 0.24, *p* < 0.001) independently predicted higher child safety concern, whereas increasing parental age was associated with slightly lower concern (*β* = −0.02 per year, *p* < 0.001). These effects are modest in magnitude and should be interpreted as exploratory given the approximately interval treatment of ordinal scores.

#### 3.2.3 Domain 3. Parental functional impairment and disruption of daily routines

Addressing RQ2 and RQ4: marked difficulty (scores 4–5) in sending the child back to school was reported by 38.7%, whereas 25.9% reported this was easy/very easy (scores 1–2). Difficulty going to work was reported by 22.1%, and difficulty allowing the child to spend time with peers outside the home by 21.8%. Difficulty resisting the urge to constantly cheque for event updates was reported by 34.4%. Mothers reported greater difficulty sending the child back to school than fathers (*χ*^2^ = 20.46, *p* < 0.001, Cramer’s *V* = 0.103, small effect), and more often reported difficulty resisting constant information checking (*χ*^2^ = 25.62, *p* < 0.001, *V* = 0.12, small effect). Detailed frequencies are in Table 3.

Spearman correlation analyses demonstrated a strong association between concern for child safety and difficulty sending the child back to school (*ρ* = 0.55, *p* < 0.001), as well as moderate associations with difficulty maintaining daily routines (*ρ* = 0.34, *p* < 0.001) and engaging in enjoyable activities (*ρ* = 0.32, *p* < 0.001).

#### 3.2.4 Domain 4. Parental cognitive–emotional processing

Almost half of the parents reported difficulty in not thinking that their child could become a victim at school. Difficulty (scores 4–5) not thinking that the child could become a victim at school was reported by 48.0%, whilst 19.3% found it easy/very easy (scores 1–2). In contrast, most parents reported that it was easy/very easy (scores 1–2), not thinking that their child could perpetrate an attack (73.1%), with 14.9% reporting difficulty (scores 4–5). Explaining to a child that killing is not normal was easy/very easy for 81.0%, whereas 9.1% reported substantial difficulty (scores 4–5). Detailed frequencies are in Table 3.

Mothers reported higher difficulty scores than fathers for cognitive–emotional items (Mann–Whitney *U*, *p* < 0.001, *r* = 0.15, small effect), consistent with the gender pattern observed in Domains 2 and 3. In contrast, the majority of parents found it easy not to think that their child could perpetrate an attack, with no meaningful gender differences.

Concern for child safety was moderately correlated with intrusive thoughts about victimization (*ρ* = 0.48, *p* < 0.001, moderate effect) and weakly-to-moderately correlated with difficulty resisting checking behaviours (*ρ* = 0.30, *p* < 0.001). These associations underscore the cognitive dimension of threat appraisal: parents who perceived greater danger were more prone to intrusive thinking and information-seeking behaviours. Full inferential results are presented in Table 4.

**Table 4:** Summary of inferential analyses.

Analysis	Variables/outcome	Test	Statistic	*p*-value	Effect size/OR (95% CI)
Gender differences (mothers vs. fathers)					
Parent age	Mothers vs. fathers	Welch *t*-test	*t* = −8.68	<0.001	*d* = −0.63 (large)
Child safety concern	Mothers vs. fathers	Mann–Whitney *U*	*U* = 259,472.5	<0.001	*r* = 0.15 (small)
Personal safety concern	Mothers vs. fathers	Mann–Whitney *U*	*U* = 252,948.5	<0.001	*r* = 0.13 (small)
Difficulty sending child to school	Mothers vs. fathers	*χ* ^2^	*χ*^2^ = 20.46	<0.001	*V* = 0.103 (small)
Difficulty resisting information checking	Mothers vs. fathers	*χ* ^2^	*χ*^2^ = 25.62	<0.001	*V* = 0.12 (small)
Cognitive–emotional processing items	Mothers vs. fathers	Mann–Whitney *U*	*p* < 0.001	<0.001	–
Correlational analyses (spearman ρ)					
Child safety concern × school difficulty	Ordinal association	Spearman *ρ*	*ρ* = 0.55	<0.001	Strong
Child safety concern × routines difficulty	Ordinal association	Spearman *ρ*	*ρ* = 0.34	<0.001	Moderate
Child safety concern × enjoyable activities	Ordinal association	Spearman *ρ*	*ρ* = 0.32	<0.001	Moderate
Child safety concern × intrusive thoughts	Ordinal association	Spearman *ρ*	*ρ* = 0.48	<0.001	Moderate
Child safety concern × checking behaviour	Ordinal association	Spearman *ρ*	*ρ* = 0.30	<0.001	Weak-to-moderate
Residence differences					
Child safety concern by residence	Urban/Suburban/Rural	One-way ANOVA	*F*(2,1924) = 2.64	0.071	*η*^2^ = 0.003 (negligible)
ANCOVA: predictors of child safety concern					
Mother vs. father	*β* coefficient	ANCOVA	*β* = 0.53	<0.001	Small-moderate
Urban residence	*β* coefficient	ANCOVA	*β* = 0.24	<0.001	Small
Parent age (per year)	*β* coefficient	ANCOVA	*β* = −0.02	<0.001	Negligible
Prior diagnosis and post-event worsening					
Prior diagnosis × child MH worsening	Binary association	*χ* ^2^	*χ*^2^ = 27.02	<0.001	*V* = 0.12 (small)
Logistic regression: difficulty sending child to school (scores 4–5 vs. 1–3)					
Child safety concern	Per 1-point increase	OR	OR = 2.42	<0.001	95% CI 2.11–2.77
Personal safety concern	Per 1-point increase	OR	OR = 1.19	<0.001	95% CI 1.09–1.30
Parent age	Per year increase	OR	OR = 0.98	0.016	95% CI 0.96–0.99
Parent gender	–	OR	ns	ns	–
Household structure	–	OR	ns	ns	–
Residence type	–	OR	ns	ns	–
Prior child diagnosis	–	OR	ns	ns	–
Model fit	Pseudo-*R*^2^ = 0.18				

ns = not significant (*p* > 0.05). OR = odds ratio from multivariable logistic regression. Effect size benchmarks (Cohen, 1988): *r*, *V*: small ≈ 0.10, medium ≈ 0.30; *d*: small ≈ 0.20, medium ≈ 0.50, large ≈ 0.80; *η*^2^: negligible <0.01; *ρ*: weak-to-moderate 0.20–0.39, moderate 0.40–0.59, strong ≥0.60. Pseudo-*R*^2^ = Nagelkerke *R*^2^.

#### 3.2.5 Domain 5. Child*’s* emotional and behavioural responses

Addressing RQ2: most parents (48.1%) reported no observable emotional or behavioural changes in their child. When changes were reported, the most common patterns were sadness (21.9%) and fear (20.3%). Observed self-harm behaviour was extremely rare (0.1%). Worsening in child mental health problems was relatively uncommon overall but showed a statistically significant association with pre-existing diagnoses (see Domain 1).

Worsening in functional domains was reported for learning (8.5%), school achievement (7.3%), sleep (7.9%), and child mental health problems (6.3%). Any child justification of the perpetrators’ actions was extremely uncommon (0.73%).

No significant differences were observed across residence types for most child outcome variables, and justification of perpetrators’ actions was extremely rare (<1%), precluding further inferential testing.

#### 3.2.6 Domain 6. Timing and course of child symptoms

Addressing RQ2: among respondents who provided timing information, the most frequent response was that parents could’*t* recall when changes started (*n* = 295). When timing was reported, onset was most commonly within the first three days (*n* = 195; 64.4% of those reporting onset), followed by 4–7 days (*n* = 56; 18%), 7–14 days (*n* = 20; 6.6%), 14–28 days (*n* = 12; 4%), and after 28 days (*n* = 10; 3.3%). The most frequently indicated initial symptom categories included sadness/anhedonia, anxiety/fear, and intrusive thoughts.

#### 3.2.7 Domain 7. Help-seeking behaviour and perceived access to care

Addressing RQ5: post-event help-seeking was uncommon: 3.8% sought professional help for the child (*n* = 73), and 2.9% sought professional help for themselves.

Among those who sought help for the child, the most frequently selected primary reason was fear of missing something despite the absence of symptoms (39.7% of help-seekers), followed by “other” (24.7%) and symptom worsening (11.0%). Perceived access to phone or video psychological support was rated as not available at all by 21.6% of respondents and as only partially available by 46.7%. Maintaining contact with family and friends after the event was easy or very easy for 74.5% of parents. Frequencies are provided in Table 5.

**Table 5:** Help-seeking behaviour and perceived access to psychological support (*N* = 1,927).

Indicator	*n*	%
Post-event professional help-seeking		
Sought professional psychological/psychiatric help for child	73	3.8
Sought professional psychological/psychiatric help for self	55	2.9
Primary reason for seeking help for child (among help-seekers, *n* = 73)		
Fear of missing something despite absence of symptoms	29	39.7
Symptom worsening	8	11.0
Other reason	18	24.7
Remaining reasons (combined)	18	24.6
Perceived access to phone/video psychological support		
Not available at all (score 1)	417	21.6
Partially available (score 2)	899	46.7
Sufficiently available (score 3)	342	17.8
Missing/not answered	269	14.0
Maintaining contact with family/friends after event		
Easy or very easy (1–2)	1,436	74.5
Neither easy nor difficult (3)	320	16.6
Difficult or completely impossible (4–5)	171	8.9

Percentages for reasons for help-seeking are calculated from help-seekers (*n* = 73). Percentages for access and social contact items are calculated from the full sample (*N* = 1,927). Missing values for the access item may reflect item skip logic or non-response.

Addressing RQ4: in multivariable logistic regression predicting marked difficulty sending the child back to school (scores 4–5 vs. 1–3), child safety concern emerged as the strongest independent predictor (OR = 2.42 per one-point increase, 95% CI 2.11–2.77, *p* < 0.001), indicating that each unit increase in concern was associated with a 142% increase in the odds of marked difficulty. Personal safety concern was also independently associated (OR = 1.19, 95% CI 1.09–1.30, *p* < 0.001). Parent age showed a small protective effect (OR = 0.98 per year, 95% CI 0.96–0.99, *p* = 0.016). Parent gender, household structure, residence type, and prior child diagnosis were not significant independent predictors in the full model. The model explained 18% of the variance in the outcome (pseudo-*R*^2^ = 0.18). Full model statistics are presented in Table 4.

## 4 Discussion

### 4.1 Pre-event under-recognition of child mental health needs

Prior to the shootings, the vast majority of parents reported that their children had no formal psychiatric diagnosis, and only a small proportion reported psychological difficulties, most commonly anxiety-related problems (30). These rates are markedly lower than epidemiological estimates from both international and Serbian studies, which consistently report substantially higher prevalence of anxiety and depressive symptoms among school-aged children and adolescents (24–26). Our findings are not directly comparable to epidemiological screening estimates, but the discrepancy may suggest systematic under-recognition of child mental health difficulties or under-reporting of child mental health difficulties in this sample. Several explanations warrant consideration: stigma associated with psychiatric diagnoses, limited access to specialist assessment particularly outside major urban centres, insufficient parental awareness of subclinical difficulties, normalisation of distress as a developmental variation, and retrospective parental reporting bias.

Particularly concerning is the finding that a substantial number of parents did not inform school professionals about known child mental health difficulties. Given that the presence of untreated mental health difficulties, alongside other contextual risk factors, is associated with elevated risk in school violence contexts (27), insufficient family–school communication represents a potential gap in preventive frameworks. Although mental illness alone does not cause school violence (13), failure to identify and support vulnerable children may limit opportunities for mental health supportive interventions.

### 4.2 Parental functional disruption and threat perception after the shootings

Following the events, parents reported pronounced functional impairment and emotional distress. Difficulty sending children back to school emerged as a central outcome, affecting nearly 40% of parents and showing the strongest association with concern for child safety (OR = 2.42, 95% CI 2.11–2.77). Concern was disproportionate to objective exposure where all families in the study sample lacked direct or indirect involvement in the shootings. Schools, institutions ordinarily associated with safety and predictable routine, appear to have been perceived as loci of danger, triggering avoidant behaviour even among families with no proximal connection to the events. This interpretation is supported by the observation that parents expressed markedly less concern regarding children’s activities outside the school context. Consistent with Family Stress Theory (21, 22), the mass school shooting placed demands on the family system that exceeded routine coping resources, generating an accumulation of concurrent stressors: anxiety about children’s safety, disrupted routines, and the burden of explaining collective trauma to children. Our findings support this model: parents who appraised schools as dangerous were more likely to experience disruptions across multiple functional domains simultaneously. The strong correlation between safety concern and school-return difficulty (*ρ* = 0.55) is consistent with the hypothesis that appraisal-driven avoidance may represent one possible pathway linking threat perception to functional impairment in non-exposed parents, though this cannot be established from cross-sectional data alone. These patterns are consistent with cognitive models of trauma (20) and similar patterns have been documented in prior studies, where mass school violence disrupted parental assumptions about institutional protection and predictability, even among non-exposed populations (5–7).

Gender differences were consistent across domains, with mothers reporting higher levels of concern and functional difficulty than fathers. Although effect sizes were modest, these findings align with prior research indicating gender differences in threat appraisal, caregiving responsibility, and emotional processing following collective trauma (28). Our results extend this finding to non-exposed parents, suggesting that gender-differentiated responses to community-level mass violence may be a robust pattern across exposure levels.

### 4.3 Cognitive–emotional processing and disruption of routines

A substantial proportion of parents reported difficulty controlling intrusive thoughts and compulsive information checking, reflecting heightened arousal and vigilance characteristic of acute stress responses (27). These behaviours were moderately to strongly correlated with functional impairments, such as difficulty maintaining routines and engaging in enjoyable activities, indicating that increased arousal and vigilance following the shootings may directly lead to disruptions in daily functioning (5), though the cross-sectional design precludes any causal or mechanistic interpretation. Such patterns have been documented following mass trauma and linked to sustained media exposure in prior studies (14).

Notably, a non-negligible proportion of parents reported difficulty explaining to their child that killing is morally unacceptable or helping the child verbalise emotions related to the event. Given the average age of children in the sample, these findings raise important questions about parental emotional availability, coping capacity, and mentalization under conditions of collective threat (29). Similar difficulties in parental emotional processing and communication have been observed in the aftermath of other mass violence events, where parental distress interfered with supportive parent–child interactions (8).

### 4.4 Child’s emotional and behavioural responses: parent-observed changes with identifiable vulnerability

Although nearly half of parents reported no observable changes in their children, sadness and fear were the most frequently noted emotional reactions, consistent with previous findings in non-exposed populations (10, 12). Severe outcomes were rarely observed by parents, suggesting that most children did not show parent-reported emotional or behavioural changes following the event. Parent-reported observations of emotional states, cannot be equated with psychiatric deterioration or disorder. Within these constraints, the pattern suggests an absence of parent-observed changes at the population level, though this cannot be equated with resilience in the clinical sense. Nonetheless, children with pre-existing mental health difficulties were significantly more likely to show post-event worsening, supporting stress–vulnerability model (15) and highlighting the importance of targeted monitoring and support for this identifiable at-risk subgroup.

### 4.5 Help-seeking behaviour and perceived barriers to support

Despite elevated parental distress, utilisation of formal professional mental health services remained strikingly low (3.8% for the child, 2.9% for self). It is important to distinguish between different levels of support need when interpreting these figures. Formal psychological or psychiatric treatment represents only one component of a broader support continuum that includes psychoeducation, school-based psychosocial support, and community-level crisis outreach. The low rates observed likely reflect barriers to formal treatment specifically, including stigma, access limitations, and normalisation of distress, rather than a global absence of need. Most affected parents may not require clinical intervention but could benefit substantially from accessible, non-stigmatising psychoeducation and school-based programmes. This distinction has significant implications for the design and targeting of post-crisis responses. The discrepancy between distress levels and formal help-seeking is consistent with findings from other school shotings (Columbine, Parkland, Sandy Hook) and mass violence contexts (10, 24, 25, 29), and many parents additionally reported limited or absent perceived access to psychological or psychiatric support, whilst others appeared to normalise their distress as an expected parental reaction.

Evidence from broader population studies confirms that the psychological impact of mass shootings extends well beyond directly affected individuals: increased antidepressant use has been documented in communities located near school shootings (6). The impact of such events often radiates outward from the epicentre, affecting direct victims first, followed by families, communities, and broader society (7). The present findings are consistent with this concentric model of trauma impact, demonstrating that parents with no direct or indirect connection to the events nonetheless reported substantial distress and functional disruption.

Mental health stigma remains pronounced in Serbia, where psychological distress is frequently attributed to personal weakness and professional help-seeking is culturally less normative than family-based coping (24). Public trust in educational and healthcare institutions was substantially tested by the societal response to the 2023 shootings (31). The events triggered mass public protests and prolonged media saturation that may have amplified collective anxiety. Research following the Utøya massacre found that parents avoided seeking support partly because they perceived themselves as a burden during a time of collective mourning (25), a dynamic that may resonate in the Serbian post-shooting context. These contextual factors likely amplified barriers to help-seeking beyond what distress levels alone would predict, and should be considered in cross-national comparisons of help-seeking rates.

### 4.6 Strengths and limitations

A major strength of this study is the large nationwide sample (*N* = 1,927) and the deliberate exclusion of directly and indirectly exposed families, which permits focused examination of community-level psychological impact. The large sample size provides adequate statistical power for inferential. The multi-domain reporting structure allows characterisation of parental responses across cognitive, emotional, functional, and behavioural dimensions. Analytical domains represent a theoretically informed consolidation of the original instrument structure and should be considered exploratory rather than confirmatory.

Several limitations warrant explicit acknowledgement. First, all data were self-reported and cross-sectional, precluding causal inference and introducing susceptibility to recall and social desirability biases. Second, the study used non-probability sampling in both phases, precluding calculation of a meaningful response rate or margin of error and limiting generalisability. Third, the sample is substantially dominated by mothers (86.4%), which constrains representativeness with respect to fathers and limits the power of gender comparisons. Fourth, pre-event child mental health characteristics were assessed retrospectively, making it difficult to disentangle true pre-event functioning from post-event reinterpretation. Fifth, the measurement instrument was developed specifically for this study and has not been externally validated. Sixth, child outcomes were assessed exclusively through parental report, without corroboration from child self-report, teacher ratings, or clinical assessment. Seventh, the two study phases involved independent samples with different recruitment frames. Eighth, the absence of a pre-event baseline or matched comparison group prevents attribution of observed responses specifically to the shooting events. Ninth, ordinal variables treated as approximately interval in ANOVA and ANCOVA secondary analyses should be interpreted with caution. Primary conclusions rely on nonparametric methods. Finally, the unique circumstances of the 2023 event in Serbia. The mass shooting at the Vladislav Ribnikar elementary school (May 3) was followed the next day by another shooting at a different location (May 4) and the killing of school children, but without fulfilling the definition of a school shooting. Although respondents who reported direct or indirect exposure to either event were excluded from the analyses, the temporal proximity of the two incidents may have made it difficult for some parents to separate their emotional and cognitive responses, which may be a potential source of response bias and response contamination. Such circumstances cannot be fully controlled for in this design and should be taken into account when interpreting the findings.

## 5 Conclusions and implications

Study provides evidence that non-exposed parents reported significant levels of safety concern, emotional distress, and functional impairment following the first mass school shooting in Serbia. Concern for child safety was pervasive, with the majority of parents reporting moderate-to-high levels, and marked difficulty in sending children back to school constituted the most prominent functional outcome. Safety concern emerged as the strongest independent predictor of school-return difficulty. Gender differences were consistent across all domains, with mothers reporting significantly higher burden than fathers. Children were largely perceived as resilient, but those with pre-existing psychiatric diagnoses were significantly more likely to show parent-reported post-event worsening, consistent with a stress–vulnerability framework. This subgroup showed substantially lower rates of formal diagnosis than epidemiological estimates would suggest, raising concern about possible under-recognition prior to the event, though the present study did not independently assess undiagnosed difficulties. Post-event utilisation of formal mental health services remained low, despite widespread distress, reflecting access barriers, cultural inhibitors to help-seeking, and limited perceived availability of support. These findings point to a gap between the community-level psychological impact and the capacity of existing mental health infrastructure to respond to it.

The results indicate that effective post-crisis response requires proactive, school- and community-based outreach targeting non-exposed parents of school-age children, particularly mothers, who reported significantly greater distress and functional impairment than fathers. Selective monitoring and targeted support are warranted for children with pre-existing mental health vulnerabilities, who showed higher rates of post-event worsening. The findings also highlight the importance of encouraging open communication between parents and school professionals regarding children’s mental health difficulties, as a substantial proportion of parents in this sample did not share relevant information with schools prior to the event abot children mental health problems. Finally, the findings underscore the need for national-level community mental health preparedness frameworks that function as a permanent component of the public health infrastructure, rather than as reactive measures. These conclusions should be interpreted in light of the study’s methodological limitations.

## Data Availability

The raw data supporting the conclusions of this article will be made available by the authors, without undue reservation.
